# Which of the 37 Plates Is the Most Mechanically Appropriate for a Low-Neck Fracture of the Mandibular Condyle? A Strength Testing

**DOI:** 10.3390/jcm12216705

**Published:** 2023-10-24

**Authors:** Jakub Okulski, Marcin Kozakiewicz, Michał Krasowski, Rafał Zieliński, Tomasz Wach

**Affiliations:** 1 Department of Maxillofacial Surgery, Medical University of Lodz, 113 Żeromskiego Str., 90-549 Lodz, Poland; jakub.okulski@gmail.com (J.O.); marcin.kozakiewicz@umed.lodz.pl (M.K.); bkost@op.pl (R.Z.); 2 Material Science Laboratory, Medical University of Lodz, 251 Pomorska Str., 92-213 Lodz, Poland; michal.krasowski@umed.lodz.pl

**Keywords:** osteosynthesis plates, open reduction internal fixation, mandible condyle fractures, MEF, PDF

## Abstract

(1) Background: The mandible is the most frequently injured component of the facial skeleton, with 25–45% of mandibular fractures involving the condylar process. This study aims to mechanically compare which plates are most suitable for use in low-neck fractures of the condyle. (2) Methods: Polyurethane mandibular models with simulated low-neck fractures were tested using 37 distinct plate designs. These plates were fabricated from 1 mm thick, grade 23 titanium sheets. The models were then subjected to force tests on a strength machine, and the correlation between applied force and fracture displacement was recorded. (3) Results: For low-neck fractures, XCP side-dedicated 3+5 and ACP-T plates demonstrated strength comparable to that of two straight plates, the current gold standard in osteosynthesis. (4) Conclusions: The Mechanical Excellence Factor (MEF) introduced by the authors provides a more accurate metric for theoretically predicting a plate’s mechanical strength compared to the Plate Design Factor (PDF). Eight plate characteristics were utilized to calculate the MEF. Employing the MEF allows for rapid, preliminary validation before undertaking strength tests. Furthermore, the findings of this study can guide the selection of the most durable plate designs for subsequent fatigue testing.

## 1. Introduction

Individuals are susceptible to various types of injuries, including traffic accidents, workplace incidents, domestic mishaps, and physical assaults. One anatomical region that is frequently subjected to injury and poses challenges for effective fracture management is the facial area, particularly the mandible. Unlike the long bones in the limbs, facial bones have unique shapes and serve multiple functions: they define facial contours, protect the brain, and house organs responsible for sight, hearing, and the initial segments of the respiratory and gastrointestinal tracts.

The mandible is the most frequently injured component of the facial skeleton, with 25–45% of mandibular fractures involving the condylar process [1,2,3]. Among these, fractures of the condylar base account for 54%, while fractures of the condylar neck constitute 16%, with an equal distribution between low- and high-neck fractures—each at 8% [3].

The market offers a multitude of plate designs for the osteosynthesis of low-neck fractures in the mandibular condyle. However, the existing literature does not conclusively identify the most effective dedicated plate for such fractures [4,5,6,7,8]. It is worth noting that newer plates specifically designed for surgical treatment of this fracture type have been introduced recently [9,10,11,12,13].

In the context of ideal osteosynthesis, two principal lines are considered optimal for plate placement. The first runs parallel below the mandibular notch and aims to restore the tensile forces in the condylar region. The second runs parallel to the posterior edge of the mandibular branch and serves to maintain sagittal plane reduction by counteracting rotational (axial plane) and bending (frontal plane) stresses that arise during mastication [14].

The objective of this study is to mechanically compare the suitability of various plates used in low-neck fractures of the mandibular condyle.

## 2. Materials and Methods

We identified 51 plate designs specifically created for osteosynthesis of mandibular condyle fractures, from both various manufacturers, such as KLS Martin, ChM, Medartis, Synthes DePuy, and Global D, and our own prototypes at the Medical University of Lodz. For low-neck fractures, 37 plates were used, owing to their specific dimensions. These were designed by the authors using CAD software (SolidWorks) under the 2.0 system. We opted for in-house design because many of the plates available on the market did not meet our criteria: some were sample designs tested only using Finite Element Methods (FEMs), while others were original designs unavailable for purchase. The plates were laser-cut from grade 23 titanium medical sheet metal with a thickness of 1 mm. This grade is closely related to grade 5, but offers enhanced ductility and fracture toughness due to its lower concentrations of oxygen, nitrogen, and iron [15].

For biomechanical testing, we utilized polyurethane bone models, conforming to the standards set by the literature and the American Society for Testing and Materials [16]. These polyurethane mandibular models were standardized and featured a uniform internal structure, closely mimicking the properties of human spongy bone. Polyurethane mandibles from Sawbones (Vashon, WA, USA; density 0.16 g/cc, compression modulus 58 Mpa) served as the testing models [9,17,18,19,20,21].

According to Kozakiewicz’s classification, mandibular condyle fractures fall into three categories: base fracture, low-neck fracture, and high-neck fracture. Our study primarily followed the AO Foundation classification, which is more widely recognized [22]. In each mandibular model, a single cut was made on both condylar processes to simulate a low-neck fracture.

The fractured condylar (proximal) and ramus (distal) sections were rejoined using a plate. Holes for the plate were drilled using a calibrated 1.5 mm drill bit, and the plate was then fastened with certified, self-tapping titanium screws measuring 2.0 mm in diameter and 6.0 mm in length. This process was repeated seven times for each type of plate.

**Figure 1:**
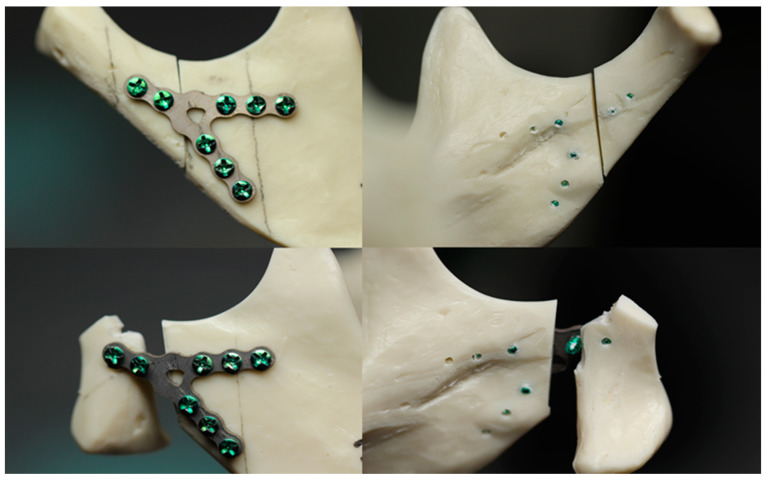
Mandible model before and after testing.

To mimic natural masticatory forces on the temporomandibular joint, the condyle was oriented at angles of 15° in the sagittal plane and 10° in the frontal plane. For this, a customized test base was constructed in the shape of a truncated cuboid. The mandibular models were firmly secured to this base. Forces were applied to the condylar articulation in upward, forward, and medial directions by a machine piston. This setup aimed to simulate as closely as possible the muscle forces acting on the condylar process during mastication (Figure 1).

**Figure 2:**
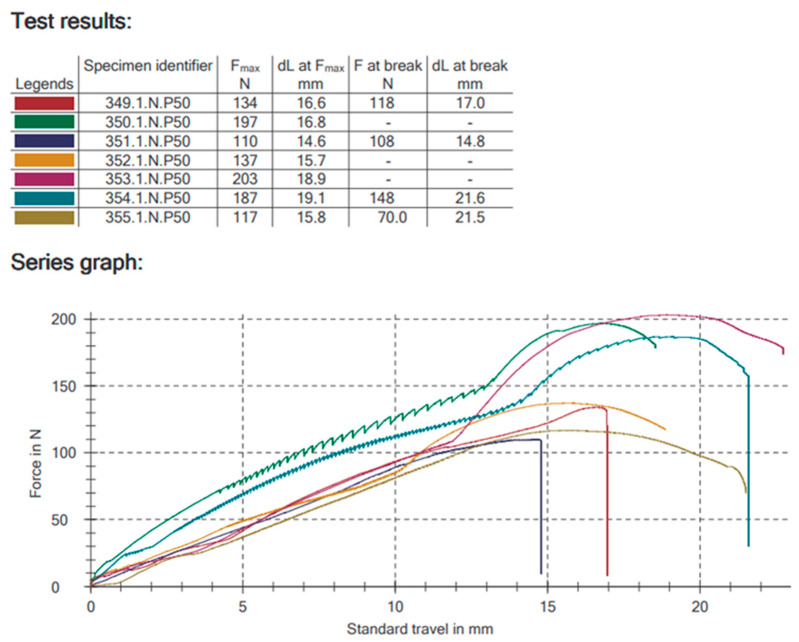
Graph from testXpert II V3.31. The graph shows the relationship between the force applied to the condylar process and its displacement. Other explanations in the text.

All tests were carried out by the authors at the Material Science Laboratory of the Medical University of Lodz, Poland. For the strength tests, a Zwick Roell Z020 universal testing machine was used (Zwick-Roell, Ulm, Germany). The initial applied force to the condyle was 1 N and then increased according to the piston travel down of 1 mm per minute. The test was terminated when a plate fractured, a condyle broke, a screw pulled out, or the deformation of the system was so severe that the condyle rested against the base of the machine. Instron software dedicated to the machine (testXpert II V3.31, Zwick Roell, Ulm, Germany) was used to record the results. The relationship between the applied force (F_max_ [N]) and the displacement of the piston (dL at F_max_ [mm]), the load when permanent deformation occurred F_max_ [N]), the maximum load at fracture (F at break [N]), and the displacement of the piston during the maximum load at fracture (dL at break [N]) were recorded (Figure 2). The maximum force required to displace the condyle by 1 mm in the entire study group was 15.23 ± 3.53 N.

The following features derived from the designs of the plates were noted: Number of Screws in Ramus, Number of Screws in Condyle, Total Fixing Screw Number, Height [mm], Width [mm], Plate Surface Area [mm^2^], % Round Holes (i.e., the percentage of circular holes in the plate among all holes designed in the plate), Number of Oval Holes in Plate (i.e., the number of oval holes designed in the plate), Oval Holes Share (the ratio of the number of oval holes for screws to the number of round holes designed in the plate).

The PDF (Plate Desing Factor) [23] and MEF (Mechanical Excellence Factor) [21] previously proposed by the authors were calculated for each plate (Table 1). PDF is a coefficient, for which four plate variables are used to calculate it: Height, Width, Plate surface area, and Total fixing screws number. The PDF is described by the following formula:

Plate Design Factor = 0.850954 × Height (mm) + 0.846751 × Width (mm) + 0.936732 × Plate surface area (mm^2^) + 0.848039 × Total fixing screws number, (1)

MEF is a coefficient, for which eight plate variables are used to calculate it: Height, Width, Plate surface area, Total fixing screws number, Number of Screws in Condyle, % Round Holes, Number of Oval Holes in Plate, and Oval Holes Share. The MEF is described by the following formula:

(2) $$\mathrm{Mechanical}\;\mathrm{Excellence}\;\mathrm{Factor}=\sqrt{0.930016\times Improvement\;Component-0.930016\times Deteriorating\;Component},$$

where:

Factor 1 = Improvement Component = 0.924362∙Total Fixing Screw Number + 0.708092∙Number of Screws in Condyle + 0.804335∙Height [mm] + 0.802964∙Width [mm] + 0.752599∙Plate Surface Area [mm^2^]+ 0.189877∙% Round Holes − 0.0300946∙Number of Oval Holes in Plate − 0.11967∙Oval Holes Share, (3)

and

Factor 2 = Deteriorating Component = −0.108505∙Total Fixing Screw Number − 0.109∙Number of Screws in Condyle − 0.0375889∙Height [mm] − 0.0761193∙Width [mm] − 0.127803∙Plate Surface Area [mm^2^] − 0.940781∙% Round Holes + 0.97506∙Number of Oval Holes in Plate + 0.980953∙Oval Holes Share, (4)

The statistics analysis was performed in Statgraphics Centurion 18 (Statgraphics Technologies Inc. The Plains City, Warrenton, VA, USA). The ANOVA or Kruskal–Wallis test was applied for between-design comparisons. Independent χ^2^ tests were used to test the categorical variables. The relationship between the two quantitative variables was assessed by linear regression analysis. The indication of the best plate design was made based on objective description. A *p*-value less than 0.05 was considered statistically significant.

## 3. Results

Table 1 presents the test results for each of the 37 plate models. The average force required to displace the condylar process fixation by 1 mm was 7.26 ± 3.61 N, with a median of 6.56 N. This value is significantly lower than that for the intact mandibular model, which measured 28.33 ± 3.16 N (Mann–Whitney (Wilcoxon) test; *p* < 0.001).

**Figure 3:**
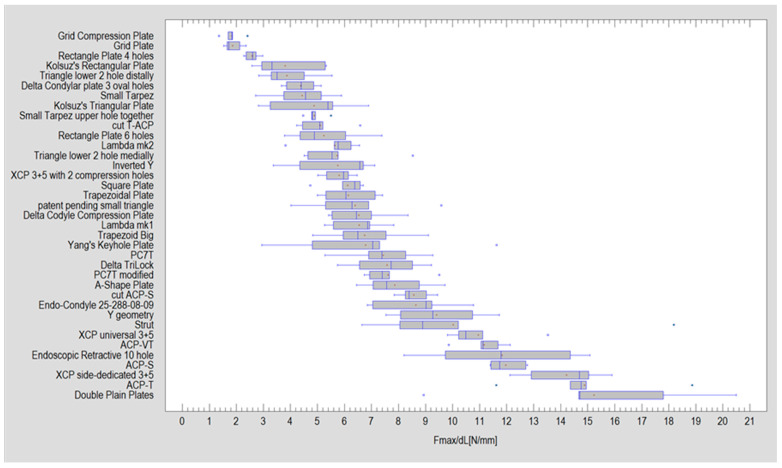
Experimental results for each plate design. Forces needed for one-millimeter displacement of the fixed fragments: mean (red cross), median (thin vertical line inside the grey box). The blue points are Fmax/dL for a given plate which are outside the standard deviation.

Significant variations were observed in the amount of force required to displace the fixation by 1 mm across all tested plate designs (Kruskal–Wallis test; *p* < 0.001). The highest value was recorded for dual-plate osteosynthesis using straight plates, measuring 15.23 ± 3.53 N. However, we found that fixations using ACP-T (Plate 23) or XCP side-dedicated 3+5 (Plate 18) were statistically as strong as those using Double Plain Plates osteosynthesis (see Figure 3 and Table 2).

**Figure 4:**
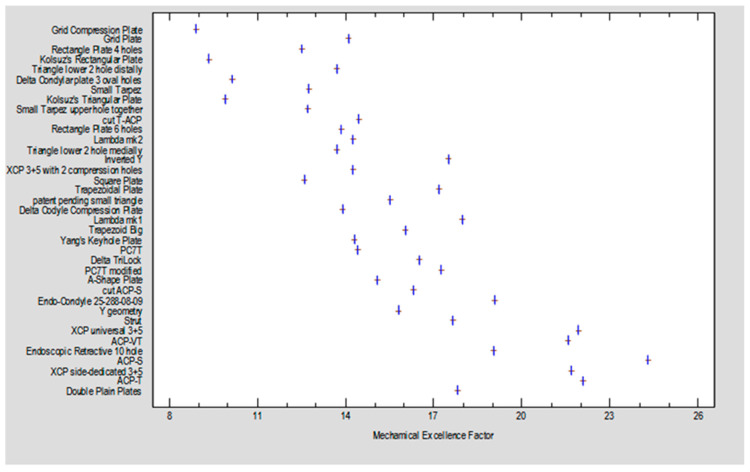
Relationship between plate and Mechanical Excellence Factor (MEF).

Figure 4 illustrates the relationship between the plate designs and the Mechanical Excellence Factor (MEF), denoted by thick vertical lines on the graph. A general trend shows that plate rigidity increases with a higher MEF value.

**Figure 5:**
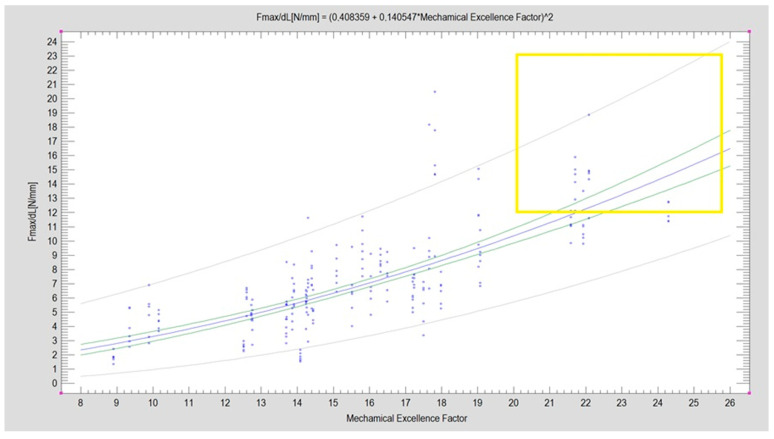
Relationship of cumulative evaluation of plate construction (Mechanical Excellence Factor, MEF) to resistance to displacement force of fixated fragments of mandibular condylar process low-neck fracture (*p* < 0.001). Explanation in the text.

Plate designs with an MEF greater than 20 are considered promising, while those achieving a force sufficient to displace segments by 1 mm above 12 N are categorized as strong (see Figure 5). The dataset encompasses all experimental results collected (259 experiments). The two characteristics have a moderately strong correlation (CC = 0.77), and the mathematical model accounts for a significant portion of the observations (R2 = 60%). The blue line represents the regression equation plot, with the points indicating the experimental results obtained. The green lines mark the 95% confidence limits, while the gray lines indicate the prediction limits. Notably, there are designs with promising construction features (MEF > 20) and high-stability fixation capabilities (Fmax/dL > 12N), with some overlap between the two sets (indicated by a yellow box).

## 4. Discussion

The results of biomechanical testing are influenced by variations in bone density and elastic modulus [24,25], which serves as a limitation of this study. Cadaver bones appear to be the most suitable material for testing plates; however, they exhibit variability. This variability is attributable to factors such as age-related differences in the compact lamina’s thickness; the degree of bone mineralization, influenced by diet and calcium supplements; and the medications administered to patients—particularly those for osteoporosis in patients with breast or prostate cancer and bone metastases. Consequently, biomechanical tests on human mandibles lack standardization [25]. Animal studies present an alternative [26,27,28,29,30,31]. While a well-established research method, such studies often involve only a few types of plates, unlike the diverse range examined in our study. Moreover, animal mandibles differ in shape, thereby affecting stress distribution and potentially rendering the results inapplicable to human osteosynthesis and fracture healing. Finite Element Method (FEM) testing of plates is another widely accepted method in preclinical research [32]. However, this approach may produce results under overly ideal conditions that do not readily translate to clinical applications. These studies necessitate considerable expertise, funding, and time to develop a comprehensive model. Three-dimensional printing could have allowed us to create replicable models of the mandible, incorporating both compact and spongy bones. However, the need for standardization, high cost, and extensive production time led us to reject this option [33].

In our study, we opted for the most readily available material: polyurethane mandible models, commonly used in orthopedic research. This choice, however, comes with its own set of limitations, such as compromised screw retention due to the absence of a cortical bone structure, which is critical for primary stabilization during osteosynthesis.

As noted above in Table 1, there is no statistical difference among XCP side-dedicated 3+5, ACP-T, and Double Plain Plates. Despite the different shape of the plates, they are equally good. Double Plain Plates are the widely used gold standard for mandibular fracture fixation worldwide. They have one major disadvantage—they are two separate plates that need to be stabilized separately in the clinical setting. This is a difficulty during surgery because the small plate (compared to the larger XCP side-dedicated 3+5 and ACP-T plates) is more difficult to screw in, especially in procedures performed with endoscope assistance. The advantage of the XCP side-dedicated 3+5 and ACP-T inserts is that the insertion of the first screw already predetermines the position of the insert. Inserting the second screw allows us to place the splint in the correct position, and then we only need to screw in the remaining screws. On the other hand, the disadvantage of these inserts is their shape resembling the letter “A”. This makes it easy to screw them into the mandibular canal and damage the inferior alveolar nerve. In the absence of a follow-up CT scan in the operating theater, if a screw is inserted into the mandibular canal, reoperation is required to reposition the plate or insert shorter screws. In contrast, Double Plain Plates have the important advantage of being able to insert one plate close to the posterior edge of the mandibular branch and the other just below the mandibular notch, minimizing the risk of nerve damage, as the distance between the plate arms is not artificially limited by a rigid bridge between them.

Ladder plates [34] merit consideration, as they outperform mini plates in certain respects, particularly in the case of extensive fractures. The authors recommend their use for fractures at the mandibular angle. Future studies should explore their applicability to neck fractures, assessing both ease of positioning and effectiveness.

Another avenue for exploration involves resorbable plates made from materials like polylactic acid (PLLA) [35]. These could be particularly useful in pediatric cases, where a second procedure for plate removal is undesirable. Potential complications, such as growth zone restrictions or tooth development issues, could be avoided [36]. However, PLLA plates have been shown to cause local bone degradation around the fracture site, potentially impeding proper healing [35].

When choosing a plate for osteosynthesis, the anatomy of the condylar process should guide plate selection. Further investigation is warranted to determine how this anatomy affects plate performance, particularly in relation to torsional forces and associated risks.

The use of ZrO_2_ plates could also be considered, especially in patients with titanium allergies [37,38]. These plates are non-cytotoxic and non-genotoxic, provided the ZrO_2_ surface is not abraded by airborne aluminum oxide particles.

The force used to insert screws is another critical factor. Inserting dental implants with a force exceeding 40 Ncm has been associated with higher rates of marginal bone loss [39]. Excessive screw insertion force could likewise compromise the primary stability of the osteosynthesis fixation.

As shown in Table 1, osteosynthesis plates for mandibular fractures come in a variety of shapes, from straight plates, through sternum plates used singly or two at a time, through square-shaped plates, to plates whose shape was determined by mandibular biomechanics: trapezoidal, delta, inverted Y, type A, type X.

Evaluation of the influence of oval holes showed that they have an adverse effect on the rigidity of the fixation, which can be seen in the delta plate (plate 2 vs. plate 37) or XCP (plate 19 vs. plate 40). The best plates are those fixed with seven, eight, or nine screws [21].

This study aims to identify the most clinically effective wafers, but it does not account for numerous biological variables. For comprehensive findings, mechanical testing should be followed by endurance fatigue and clinical tests.

To expedite the evaluation of new osteosynthesis plate designs, the MEF factor could be utilized. This factor is an improvement over the previously proposed PDF factor, offering a more accurate preliminary estimate of a wafer’s mechanical strength.

Limitations of this study include the use of polyurethane models and specific plate designs. Our findings may not fully translate to human bone conditions or to plates made from different materials or shapes. Moreover, the study focuses on a specific type of condylar process fracture based on the Kozakiewicz classification, potentially affecting the results.

## 5. Conclusions

Among the 37 plate types examined for osteosynthesis of fractures in the lower neck of the mandibular condylar process, the Double Plain Plates, XCP side-dedicated 3+5, and ACP-T are the most mechanically reliable. Utilizing these plates minimizes the risk of plate fracture, screw extraction, and inadequate bone fixation.

## Figures and Tables

**Table 1:** Compared plated designs placed from the weakest to the strongest fixing material.

Name	Design Code	Design	H [mm]	W [mm]	S [mm^2^]	PDF	MEF	Fmax/dL [N/mm]
Grid Compression Plate	Plate 35	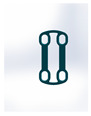	17.3	9.1	70	92	8.9	1.83 ± 0.31
Grid Plate	Plate 36	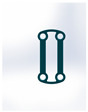	19	9.6	80	102	14.1	1.85 ± 0.29
Rectangle Plate 4 holes	Plate 32	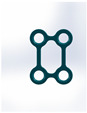	11	8.5	37	55	12.5	2.59 ± 0.23
Kolsuz’s Rectangular Plate	Plate 48	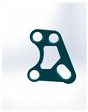	11.4	10.1	49	67	9.4	3.81 ± 1.10
Triangle lower 2 hole distally	Plate 42	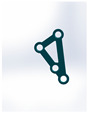	17.8	10	67	90	13.7	3.87 ± 0.90
Delta Condylar Plate 3 oval holes	Plate 37	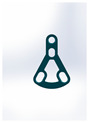	17	11.4	64	87	10.2	4.39 ± 0.52
Small Trapez	Plate 33	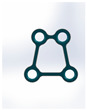	11.6	11.6	41	61	12.8	4.43 ± 1.00
Kolsuz’s Triangular Plate	Plate 47	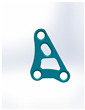	14	10.4	58	79	9.9	4.87 ± 1.42
Small Trapez upper hole together	Plate 34	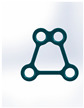	11.1	12.1	40	61	12.7	4.89 ± 0.31
cut T-ACP	Plate 44	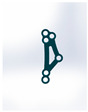	23.6	9.3	85	112	14.5	5.10 ± 0.75
Rectangle Plate 6 holes	Plate 38	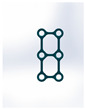	20	10	67	94	13.9	5.23 ± 1.18
Lambda	Plate 41	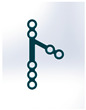	25.1	13.3	72	106	14.3	5.65 ± 0.87
Triangle lower 2 hole medially	Plate 45	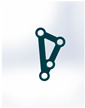	18.1	9.8	67	90	13.7	5.73 ± 1.33
Inverted Y *	Plate 28	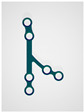	23.6	11.1	203	225	17.5	5.76 ± 1.40
XCP 3+5 with 2 compression holes	Plate 40	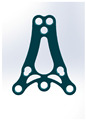	20.5	16	107	138	14.3	5.81 ± 0.49
Square Plate	Plate 31	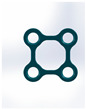	10	10	39	57	12.6	6.11 ± 0.67
Trapezoidal Plate	Plate 51	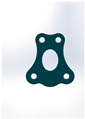	20	18	189	213	17.2	6.14 ± 0.88
Patent pending small triangle *	Plate 11	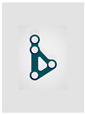	13.5	8	138	151	15.5	6.40 ± 1.70
Delta Condyle Compression Plate *	Plate 14	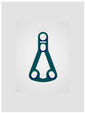	15.3	8.8	179	191	13.9	6.53 ± 0.98
Lambda Thinned *	Plate 01	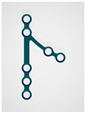	25.6	13	219	240	18.0	6.54 ± 0.87
Trapezoid Big	Plate 39	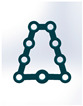	20.8	19.4	131	165	16.0	6.74 ± 1.33
Yang’s Keyhole Plate	Plate 46	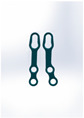	19.7	9,.9	80	106	14.3	6.79 ± 2.66
PC7T	Plate 49	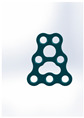	15	12,8	86	110	14.4	7.44 ± 1.27
Delta TriLock *	Plate 02	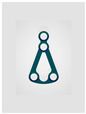	15.4	8.8	174	187	16.5	7.58 ± 1.16
PC7T modified *	Plate 03	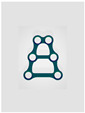	13.5	11.7	199	213	17.2	7.61 ± 0.91
A-Shape Plate	Plate 43	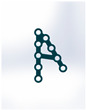	23.6	14.1	98	131	15.1	7.86 ± 1.09
Cut ACP-S *	Plate 08	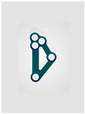	14.9	8.1	165	179	16.3	8.56 ± 0.53
Endo-Condyle 25-288-08-09 *	Plate 21	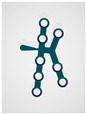	22.7	11	271	289	19.1	8.65 ± 1.35
Y geometry	Plate 50	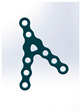	25	19	119	155	15.8	9.41 ± 1.48
Strut *	Plate 04	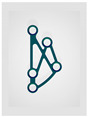	19	9.6	217	232	17.7	10.03 ± 3.76
XCP universal 3+5 *	Plate 19	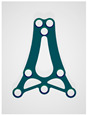	22.7	18	407	423	22.0	10.96 ± 1.22
ACP-VT *	Plate 12	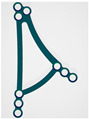	37	21	371	404	21.6	11.17 ± 0.70
Endoscopic Retractive 10 hole *	Plate 17	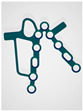	21.6	15.3	290	311	19.0	11.83 ± 2.40
ACP-S *	Plate 25	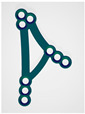	26	16.3	538	547	24.3	11.97 ± 0.57
XCP side-dedicated 3+5 *	Plate 18	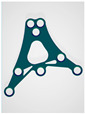	22.7	20	393	411	21.7	14.21 ± 1.30
ACP-T *	Plate 23	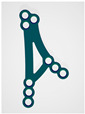	30.4	15	410	428	22.1	14.87 ± 2.11
Double Plain Plates *	Plate 20	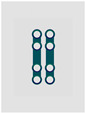	16.5	3.4	227	236	17.8	15.23 ± 3.53

* Plate designs correspond to the designs presented in an earlier publication [18]. Abbreviations: H—height, W—width, S—surface area, PDF—Plate Design Factor, MEF—Mechanical Excellence Factor.

**Table 2:** Multiple range tests for force displacing the fixation by 1 mm (Fmax/dL, N) by Plate.

Homogenous Groups ^1^	Mean Fmax/dL	Name
X	1.83	Grid Compression Plate
X	1.85	Grid Plate
XX	2.59	Rectangle Plate 4 holes
XX	3.81	Kolsuz’s Rectangular Plate
XX	3.87	Triangle lower 2 hole distally
XX	4.39	Delta Condylar Plate 3 oval holes
XX	4.43	Small Tarpez
XXX	4.87	Kolsuz’s Triangular Plate
XXXX	4.89	Small Tarpez upper hole together
XXXXX	5.1	cut T-ACP
XXXXXX	5.23	Rectangle Plate 6 holes
XXXXXX	5.65	Lambda
XXXXXX	5.73	Triangle lower 2 hole medially
XXXXXX	5.76	Inverted Y
XXXXXX	5.81	XCP 3+5 with 2 compression holes
XXXXXX	6.11	Square Plate
XXXXXX	6.14	Trapezoidal Plate
XXXXXX	6.4	Patent pending small triangle
XXXXX	6.53	Delta Condyle Compression Plate
XXXXX	6.54	Lambda Thinned
XXXX	6.74	Trapezoid big
XXX	6.79	Yang’s Keyhole Plate
XXX	7.44	PC7T
XXX	7.58	Delta TriLock
XXX	7.61	PC7T modified
XX	7.86	A-Shape Plate
XX	8.56	Cut ACP-S
XX	8.65	Endo-Condyle 25-288-08-09
X	9.41	Y geometry
XX	10.03	Strut
XX	10.96	XCP universal 3+5
XX	11.17	ACP-VT
X	11.83	Endoscopic retractive 10 holes
X	11.97	ACP-S
X	14.21	XCP side-dedicated 3+5
X	14.87	ACP-T
X	15.23	Double Plain Plates

^1^ Within each column, the levels containing X’s form a group of means, within which there are no statistically significant differences. The method that discriminates among the means is Fisher’s least significant difference (LSD) procedure. With this method, there is a 5.0% risk of labeling each pair of means significantly different when the actual difference equals 0.

## Data Availability

The data presented in this study are available on request from the corresponding author. The data are not publicly available due to an ongoing multicenter project.
